# Grievance-fueled violence can be better understood using an enactive approach

**DOI:** 10.3389/fpsyg.2022.997121

**Published:** 2022-10-19

**Authors:** Bram Sizoo, Derek Strijbos, Gerrit Glas

**Affiliations:** ^1^Threat Management Team, Netherlands Police Agency, Driebergen, Netherlands; ^2^ORCAT, Zutphen, Netherlands; ^3^Dimence, Zwolle, Netherlands; ^4^Department of Philosophy of Mind and Language, Radboud University, Nijmegen, Gelderland, Netherlands; ^5^Department of Philosophy, Faculty of Humanities, Vrije Universiteit Amsterdam, Amsterdam, Netherlands

**Keywords:** grievance-fueled violence, enactive approach, philosophy, lone actor, radicalization, terrorism

## Abstract

Understanding lone actor grievance-fueled violence remains a challenge. We believe that the concept of grievance provides an opportunity to add an engaged, first-person perspective to the assessment of lone actor extreme violence. We propose an enactivist philosophical approach that can help to understand the why and how of the pathway from grievance to violent extremism. Enactivism sees grievance as a dynamic, interpersonal, and context-sensitive construct that indicates how (potential) offenders make sense of the world they live in and how under certain circumstances it fuels violent behavior. Hence, grievance should not be understood as a given thing, but as an unfolding experience that involves sense-making through (regulation of one’s) interaction with the (social) environment. This (self-)relational and ecological understanding requires another approach than looking at demographic factors or life histories, only from an outsider’s perspective. Enactivism invites us to look at such risk factors as external indices of an ongoing process of active self-regulation and sense-making, and in some cases spiraling toward extreme violence. To understand the mindset of the offender we need to look more in depth at the processes that shape this mindset: why does this person, with this history, in this context, and at this point in time, proceed to use violence? The enactivist approach to the mind offers a complementary framework that may help us to understand the dynamics of grievance as a possible precursor to violent extremism. It also helps to appreciate why the relative unpredictability of the pathway toward lone actor extreme violence is not necessarily a sign of empirical weakness but a matter of principle due to the non-linearity of the processes involved. We end by summarizing how enactivism could contribute to the prevention of extremist violence and research and how it can help to avoid reinforcing stigmas and re-establishing a confirmation bias.

## Introduction

Despite the many examples of violent extremism by individuals acting independently, our understanding of the underlying motives is still limited. Current explanatory models were designed to comprehend and counter violent extremism but none of these are completely satisfactory as we keep being caught off guard by shocking attacks that had been maturing in the minds of offenders for some time. The challenge is to grasp why, when, and especially how the mindsets of potential perpetrators develop through interaction with their environment, to enable early and appropriate preventive interventions. According to Borum (2014) ‘mindset - a relatively enduring set of attitudes, dispositions, and inclinations - and worldview are the basis of a psychological “climate,” within which various vulnerabilities and propensities shape ideas and behaviors in ways that can increase the person’s risk or likelihood of involvement in violent extremism’ (p. 286). The question is whether we can understand concepts like mindset and worldview from the perspective of potential offenders, that is, from within instead of as third-party attributions.

Among risk factors that have been associated with violent extremism, grievance has been coined as one of the factors that could shed light on the underlying motives (McCauley and Moskalenko, 2008; Meloy and Yakeley, 2014; Hafez and Mullins, 2015; Meloy and Gill, 2016; Corner et al., 2018; Pathé et al., 2018). Grievance is a complex concept that should be studied from an interdisciplinary perspective. We suggest that an enactivist approach to mental phenomena may help to develop a more comprehensive view.

The remit of this paper is not to find the Holy Grail for preventing future lone actor attacks by means of a philosophical approach. Rather, we suggest that philosophy adds to the transdisciplinary perspective that Decety et al. (2018) consider mandatory for an understanding of the precursors of radicalization and extreme violence (Decety et al., 2018). The authors stress the need for collaboration by experts in evolutionary biology, neuroscience, psychology, anthropology, economics, and political science. We believe philosophy too has an important role to play here.

We focus on lone actors and presuppose that grievance is a useful concept to understand their extreme violence. We argue that an enactivist approach can help to understand grievance as a dynamic, interpersonal, and context-sensitive construct that indicates how (potential) offenders make sense of the world they live in and how under certain circumstances it might fuel violent behavior. The enactivist perspective suggests that instead of focusing on (potential) perpetrators and their motives from an outsider’s (third person) perspective in terms of demographic and psychosocial risk factors, we should examine what goes on in their minds given their bio-psychosocial and existential situation. Although to mental health professionals with clinical experience this may sound familiar, the enactive approach goes beyond, but does not substitute, a psychiatric formulation. For those professionals in risk assessment without clinical experience, the enactive approach may serve as a caution against the use of simplistic computational methods. We will (1) start with an analysis of the concept of grievance; (2) argue that grievance is not a root cause but can help us to understand the route to extremist violence; (3) introduce enactivism; and (4) show how this approach to grievance and radicalization might help to develop an understanding ‘from within’ that is multidisciplinary, ecologically valid, and tailored to the individual offender.

## Grievance

Grievance, according to the Cambridge Dictionary means: ‘a complaint or a strong feeling that you have been treated unfairly’ (Cambridge University Press, 2013). Grievance therefore contains two elements: it emerges through interpersonal relatedness and involves sense-making. The implicit negative affect (sense-making) associated with grievance is related to the perceived injustice inflicted by another (interpersonal relatedness). It is imaginable that grievance can lead to avoidant or violent behavior. In other words, the definition of grievance offers some ground for a temporal relationship with violent extremism.

Grievance should be distinguished from moral injury, which refers to disturbing, even traumatizing, events that bring about fundamental changes in one’s morality and personhood, leading to a re-valuation of core values. Moral injury is ‘an act of transgression that creates dissonance and conflict because it violates assumptions and beliefs about right and wrong and personal goodness’ (Litz et al., 2009, p. 698). The transgression may be caused by another person, but also by oneself (Koenig and Al Zaben, 2021). This old concept was re-invented in the seventies of the previous century to identify experiences of damage done to the conscience of American soldiers during the Vietnam war, who (had to) perform acts or failed to prevent acts that conflicted with their personal values or ethics. Much later the term was also used in the context of healthcare, the police, and (other) crisis management agencies. Grievance refers to (perceived) injustice caused by others whereas moral injury focuses on injury caused by others or oneself, leading to disruption of one’s moral self. Injustice (in cases of grievance) can of course be seen as a form of injury with an impact on the moral self. So, on conceptual grounds we may suspect that there are cases in which both concepts hold.

Grievance has been associated with violent extremism in the literature. In 2006, the U.S. Department of State (2006) noted that the grievance of an individual can be targeted by terrorists to stimulate radicalization by linking their radical group narrative to pre-existing personal questions and sentiments associated with grievances.

In other cases, the link between personal motives and perceived injustice is clearer. Adrei Zhelayabov led his terrorist group *Narodnaya Volya* (People’s Will) into making bombs, one of which killed Czar Alexander II in 1881. Zhelayabov is assumed to have been motivated by his personal suffering directly inflicted by the Czarist regime (McCauley and Moskalenko, 2008). Another example is Elliot Rodgers, who killed six and wounded 14 others in 2014 because he wanted to punish women for rejecting him, and sexually active men because he envied them. We are informed about his state of mind because he left a 133-page manifesto My Twisted World: ‘All I have ever wanted was to love women, but their behavior has only earned my hatred. I want to have sex with them, and make them feel good, but they would be disgusted at the prospect. They have no sexual attraction toward me. It is such an injustice, and I vehemently questioned why things had to be this way’ (Hoffman et al., 2020, p. 569).

In other cases, people develop a grievance because they perceive the way others whom they identify with are treated, as a personal injustice: the bigger story is connected with, imposed on, or implanted within the personal story. IS-Propaganda made effective use of this mechanism by exposing internet users to narratives of Muslim suffering, caused by Western agents (Speckhard and Ellenberg, 2020, 98). Technically, there is no direct personal victimization, nor direct interpersonal contact with a victim, but only the online exposure to the narrative. This induces a strong sense of personal injustice which may subsequently evolve into an expressed grievance and violent behavior.

We posit that, in the case of the above-mentioned identification with group victimization, the adopted grievance is preceded (and in a sense fueled) by a deep unresolved personal grievance of a different nature (e.g., abuse, neglect, bullying; Horgan, 2014, p. 85). In the literature, there is some evidence of this. Simi et al. (2016), for example, conducted in-depth interviews with past white supremacists to show that non-ideological precursors to violence were common. Of the 44 individuals that were interviewed, 37 (84%) had experienced adverse childhood conditions that can be qualified as grievances, such as physical and sexual abuse, or emotional and physical neglect (41 percent). Interestingly, the authors mention that these precursors could also have developed into general criminal violence instead of radicalization.

Grievance has been mentioned as a possible contributing factor or even likely precursor to violent extremism, among many other risk factors, but it remains unclear how this contribution should be conceptualized from a transdisciplinary point of view (Corner et al., 2018; Pathé et al., 2018). In the next section, we will first discuss traditional approaches (more in general) and then focus on the heuristic potential of an enactivist perspective to guide interdisciplinary research on grievance.

## From roots to routes

Risk assessment has progressed from attempting to predict violence from the absence or presence of a long list of risk factors, to instead, aiming to identify those in need of active monitoring or management. Corner et al. (2021) for example, reviewed the causal role of risk factors in personality, personality disorders, and psychopathy in relation to terrorism, and concluded that, whereas an overarching ‘terrorist personality’ would be an oversimplification of reality, many studies drew conclusions based on personality variables, without sound methodological or conceptual appreciation of these constructs. Therefore, thinking about risk factors as root causes of terrorism is not correct, and too simplistic use has led to confusion, as other authors have pointed out (e.g., Silke, 2009; Horgan, 2014, 98; Ward and Beech, 2015, p. 100). Failing to find a comprehensive terrorist profile and associated set of risk factors, researchers increasingly took to studying routes, not roots (Horgan, 2008; Gill and Corner, 2017). This meant documenting the personal accounts of perpetrators, and reconstructing the pathways to violent extremism, from beginning to end. Horgan (2008) argues that: ‘a clear implication of thinking about initial involvement as part of a process is that it provides a clear agenda for psychological research on terrorist behavior: an attempt to understand the decisions made by the individual at particular times within a particular social and organizational context’ (p. 90). Researchers have warned, however, against erroneously concluding that pathways to violent behavior and beliefs associated with radicalization follow a linear course, like a ‘conveyor belt’ mechanism with a predictable sequentiality and outcome, because in the literature, associations between (radical) beliefs and (radical) behavior have been shown to be weak (Wicker, 1969; McCauley and Moskalenko, 2017). Furthermore, the exploration of pathways covers different disciplines. For example, the interdisciplinary field of social neuroscience has shown that multilevel analysis of ideology and political preference corresponds with cognitive and personality signatures (Hirsh et al., 2010; Zmigrod et al., 2021), and more deeply-rooted needs and drives that have unique neurobiological correlates (Decety et al., 2018). Anthropological ethnographies made in conflict zones and among radicalized individuals illustrate that victimization (grievance) is particularly strong when sacred values are challenged, which increases the readiness to use violent extremism, as opposed to the challenge to ordinary values. These mechanisms have been shown to correlate with neuroimaging data at group level (Atran and Gómez, 2018; Hamid et al., 2019; Pretus et al., 2019). A saliant finding in the neuroimaging studies was that, along the pathway, ordinary values appeared to shift toward sacred values when participants were subjected to a social exclusion task, designed to induce a strong sense of personal injustice. In other words, the study suggests that the perception of grievance, of not being accepted in the social context, increased the readiness to use extreme violence, even when ordinary values are believed to be in jeopardy.

The Situation Action Theory (SAT) of Moral Action and Crime Causation was developed as a conceptual model to address theoretical shortcomings in terrorism research, like the misconceptions about the role of risk factors mentioned above (Wikström, 2004). SAT resembles a pathway model because it conceptualizes terrorism primarily as moral action, which develops through the interaction of an individual with his environment both as rational and experiential processes. A distinction is made between direct causes and more distal (early developmental) causes of causes. Bouhana and Wikström argue that the vulnerability to adopt a radical stance and move on to violence can be understood by the dimensions susceptibility to moral change and susceptibility to exposure to radicalizing settings (Bouhana and Wikström, 2010). An assumption of SAT is that human beings are guided by rules and that behavior does not only follow from self-interest and rational choices. Also, SAT postulates that all actions result from (1) the perception of available action alternatives and (2) the choice that a person makes. Interestingly, in SAT the subjective perception of possible alternatives (and therefore not of other alternatives) is linked to moral causes: ‘The causes of terrorism are found in the individual and environmental factors which influence a person to see an act of terrorism as an action alternative and that influences their process of choice to carry out such an act’ (Bouhana and Wikström, 2010, 54). The emotions that accompany the moral change are likely to overlap with grievance. Although SAT offers a sound and thorough model, it cannot fully explain how the interaction between the (moral) context and the individual takes place. Below we will argue that underlying moral change is a continuous and iterative process of sense-making that can be understood from an engaged perspective. Grievance can be a key constituent in this sense-making process.

In the next section, we will consider grievance—still understood as injustice inflicted by others—not as a given thing, but as a perception that involves sense-making through (regulation of one’s) interaction with the (social) environment. This (self-)relational and ecological understanding requires another approach than looking at demographic factors or life histories from an outsider’s perspective. To understand the mindset of the offender we need to look more in depth at the processes that shape this mindset: why does this person, with this history, in this context, and at this point in time, proceed to use violence, and what does the path that he has taken look like? The enactivist approach to the mind offers a framework that may help us to understand the dynamics of grievance as precursor of violent extremism.

## Enactivism

The term enactivism was introduced by Varela et al. (1991), who envisioned a new research program in cognitive science based on the idea of cognition as an essentially *active* phenomenon. In an attempt to merge cognitive science with insights from phenomenology and biology, they argued for an approach to cognition that starts with the embodied experience of organisms in the act of regulating their dealings with the environment, much like how the physical properties of the membrane of a single cell organism determine how it adapts to the properties of the surrounding fluid. Cognition was no longer conceived as passive computation in the head ‘sandwiched’ between sensory input and behavioral output taking place in the rest of the body. In place of this ‘sandwich model’ of cognition (Hurley, 1998), a picture emerged that approached cognition as an activity *in*—rather than in-between—the ongoing process of perception-action loops. Enactivism holds that perception (input) and action (output) are intertwined (‘coupled’) and the one cannot be understood without the other (Nöe, 2004; Gallagher, 2005; Thompson, 2007; Noë, 2009). Rather than sidestepping experience as an epiphenomenon to cognition, enactivism took embodied experience as a key entry point to studying cognition. And instead of taking the computer as the leading metaphor in conceptualizing cognition, it modeled cognition on the living organism as studied in evolutionary and ecological biology.

Enactivism has given rise to the so-called ‘4E’ approach to cognition (*cf.* Newen et al., 2018): mental processes and experience are *embodied* (i.e., integrated within a living body, including the brain), *embedded* in a context (from physical contexts to the social and other norm-responsive contexts), *enacted* (i.e., being shaped and created by the organisms own actions and interactions within the environment), and *extended* (i.e., mediated by socio-cultural and technological ‘scaffolds’ in the external world like social institutions, cultural narratives, sensors, smartphones, and computers). Over the last decades, several forms of enactivism have emerged: sensori-motor theory (O’Regan, 2001; Degenaar and O’Regan, 2017) phenomenologically-inspired accounts (Gallagher, 2005) variants that overlap with dynamical systems theory (Thompson, 2007; Stewart et al., 2010) and, more recently, blends with computational approaches to (network-) neuroscience and psychiatry (Ramstead et al., 2022).

For our purposes, we will follow Di Paolo et al.’s (2010) characterization of enactivism in terms of five interlocking concepts: embodiment, experience, autonomy, sense-making, and emergence. We have already touched upon embodiment and experience. Enactivism conceptualizes mental activity as an inherently *embodied* activity. Conversely, the body is not a puppet controlled by the mind/brain; rather, the body is part of an animate system that constitutes mental life, as will be illustrated in the case study below.

Second, *experience* is considered a central aspect of cognition, both methodologically and thematically. Experience forms an integral part of the study of cognition and is a guiding principle in the dialog between the various scientific disciplines involved.

Next, the notion of *autonomy* refers to fact that living organisms generate their own identity by self-sustaining and distinguishing themselves from their environment. Autonomous systems are composed of processes that actively generate and sustain their identity under precarious conditions (De Jaegher, 2013). They constantly regulate themselves and their interaction with the world to satisfy their needs and concerns created by these precarious conditions. Hence, interaction with the environment is not a matter of reactively responding to external perturbations, in the sense of selecting and producing the appropriate response to a given situation. Rather, it is an essentially active phenomenon, by which the organism actively regulates the conditions of its exchange with the environment (Di Paolo et al., 2010).

This brings us to the notion of *sense-making.* Experience, and mental activity more generally, are to be understood quite literally as a matter of actively *making* sense by regulating oneself and one’s interaction with the environment. Thus, adjusting one’s posture or position in order to get a good look is part of the process of visual perception, and writing a farewell letter is part of the process of making sense of one’s sadness or grief. Living systems generate meaning *by* their actions; they ‘enact a world’.

Lastly, the concept of *emergence* is applied to describe the formation of a novel property or process out of the interaction between parts of a system or the interaction between a system and its environment. An emergent process has its own autonomous identity and the activity required to sustain this new identity in interaction with the environment constrains and modulates the operation of underlying levels of organization.^1^ An example is the living cell: it is the result of a complex self-sustaining network of chemical transformations that at the same time influences the activity of its component chemical processes (e.g., by creating an internal milieu in virtue of its membrane). In analogy, a group of individuals may form a social system or institution with its own identity-preserving operations that constrain the (cognitive) activities of its members.

This quick-and-dirty characterization of enactivism already points toward an important contrast with some of the models of violent extremism discussed above. The behavior of human beings, and living organisms in general, is not to be conceived as a collection of reactive responses to internal or external occurrences determined by pregiven skills, traits, preference sets, and vulnerabilities. From an enactivist perspective, it is interpreted first and foremost as *acts of ongoing sense-making* whereby agents attempt to regulate their interaction with the (ecological and social) environment to satisfy their needs, concerns, and interests—thereby also regulating their physical, emotional, and cognitive states. This self-regulatory activity is constrained both by the possibilities for action available to the agent and the possibilities that the environment affords. In practical terms, the language someone masters determines what websites can be understood, and the availability of weapons makes an activity more or less likely.

An important notion to add here is that of *niche construction*. Living organisms tend to adapt their physical and social environment so as to fit their needs, thereby molding their possibilities for self-regulation in a particular direction, opening up new ways of self-regulation while constraining or downplaying the importance of others. Examples of niche construction are the building of nests or houses and the creation of working environments. Social niche construction happens when, for example, one forms new social groups or becomes a member of an (online) community. Developing a group identity and creating one’s own life narrative using socio-cultural scaffolds provided in literature, media, and so on, can be understood in terms of niche construction too: it opens up new routes for regulating one’s interaction with the environment, closing off other routes. Making sense of the world and oneself in the course of a life often involves the (co-)creation of such niches. Agent and (social) environment are structurally coupled, meaning that the interaction exerts a structuring influence on the stability and functioning on both sides of the relationship. Each subsystem (for example an agent and another person or group) acquires its own role in the interaction, simultaneously constraining and being constrained by how the interaction unfolds. In a process of dynamical co-constitution, what emerges in the interaction between agents is a novel autonomous system with a correlative niche, shaping and constraining the possibilities of the constitutive subsystems.

Participatory sense-making (De Jaegher and Di Paolo, 2007) refers to the social dimension of this concept. Participatory sense-making is ‘the coordination of intentional activity in interaction, whereby individual sense-making processes are affected, and new domains of social sense-making can be generated that were not available to each individual on her own’ (p. 497). Like in a dance where the moves of the individual dancing partners are ‘taken up’ into a larger whole (the dance itself), the social interaction between two or more agents can take on a life of its own, resulting in qualitatively new forms of sense-making that shape the sense-making activity of the individuals involved in the interaction.^2^ Applying the notion of participatory sense-making to violent extremism helps to appreciate the fact that beliefs and intentions leading to violence often cannot be understood properly without taking into account the larger context within which they took shape. Individual sense-making that evolves within a carefully crafted social niche (e.g., a family, school, or online community) can be modeled as an instance of participatory sense-making in which the collective process of sense-making heavily shapes and constrains the individual agent’s possibilities for making sense of themselves and the world.

## A case study

We will illustrate our enactivist approach to grievance-induced violence by focusing on the vignette described in Box 1 (Dylann Roof). We build our account on the description of the path toward intended violence by Allely and Faccini (2019), who intended to understand how Roof progressed on his pathway toward violence by framing the clinical findings and other critical factors in a threat assessment perspective. We followed Allely and Faccini’s description of these steps to gain even more depth by putting them into an enactivist account of how the diachronic dynamics of Roof’s sense-making and self-regulation spiral toward violent extremism.

BOX 1

Dylann Roof (1994) grew up in Columbia, South Carolina. His parents separated when he was five. He lived with his father and was mainly raised by his stepmother until she left his father in 2009. From childhood, there were concerns about his social communication skills and fixated interests. When he stopped attending classes in 2010, he had attended six schools in nine years. He started isolating himself more and more in his room, playing computer games, and using alcohol and drugs to the dismay of family members whom he increasingly rejected.

His grievance and anxiety became racial in 2013 after intense reading on the internet about black-on-white-crime, ‘Muslim gang-rapes’, and ‘Jewish control’. He wanted to ‘help the white race’ by starting a racial civil war and to be remembered for it. When he mentioned this to a friend, Roof wasn’t taken seriously.

In the three months prior to the attack, Roof was arrested three times for loitering in a shopping mall and making disturbing remarks, for possession of a forearm grip and magazines for a semiautomatic weapon, and for breaking his imposed ban from the shopping mall. He was released on all three occasions without charges.

On 17 June 2015, Dylann Roof shot nine people at Emanuel African Methodist Episcopal Church in Charleston, South Carolina, after having joined them for a prayer group session. He was apprehended by police, charged, and later pleaded guilty. Three out of five psychiatric assessments suggested that Roof had an autism spectrum disorder (ASD). He reported having had friends in the past but could only name one. He believed he was socially inadequate and had always feared being judged by others. Authorities found his website with pictures of himself with white supremacy and neo-Nazi symbols, as well as a manifesto demonstrating how he thought about other ethnic and cultural groups.

He was sentenced to nine consecutive sentences of life imprisonment without parole on state charges and sentenced to death on a federal charge after refusing to allow a psychiatric defense to be put forward.

Here, grievance is the first step in the Path to Intended Violence. Roof was a person who felt inept, socially inadequate, humiliated, criticized, and judged by others long before the assault. He thought he was physically disabled. He felt that the left side of his body was more developed and believed that this could only by attributed to a lateralized distribution of testosterone. In enactivist terms, this first phase in the development of intended violence is characterized by a deeply embodied sense of self that nourishes his sense-making from the very start. The deep feelings of inferiority were rooted in an experienced physical disability, i.e., the feelings of enlargement of the left half of his body. His sense of self was also dynamically co-constituted by his interaction with his environment. It was in the interaction with peers at school and with his family that his initial feelings of inferiority emerged. These feelings were later confirmed, validated, and put in a different context through interaction with people on the internet. This process started bottom-up with imprecise and pervasive feelings of unease, inferiority, being excluded, and being treated unfairly. Haq et al. (2020) introduce the concepts of situated affectivity and affective ‘scaffolding’ for this process. Digital communities are a rich source of narratives offering niches to accommodate anyone who may harbor feelings of exclusion, inferiority, and (presumed) injustice. The narrative functions as a scaffold that provides identity, meaning, and a purpose in life.

The next steps in the Path toward Intended Violence involve ‘ideation’, ‘research and planning’, and ‘preparation’. As explained, in order to understand his sense-making activities, the enactivist approach does not consider the individual in isolation (i.e., Roof) as the primary unit of analysis, but takes the interaction between Roof and his environment as its point of departure. The interaction between Roof and his environment is regarded as a web of relations in which the role of the actors (agents) is dynamically co-constituted by the interaction. Ideation, interpretation, and planning build forth on this interaction and are an expression of it in those who are (complementarily) involved in the interaction. In other words, in the structural coupling between the individual and the environment, the interaction between (sub) systems exerts a structuring influence on the stability and functioning on both sides of the relationship. Each subsystem (i.e., Roof and the people he interacts with) acquires its own complementary role in the interaction, their autonomy is shaped and constrained by the newly-emerged overarching relational dynamics. In this way, Roof’s self-regulation and sense-making possibilities—his identity, agency, and sense of self—develop in a certain direction, correlative with the construction of a social niche as an essential part of a dynamical process between initially Roof’s family, peers, school, and so on, and, later, digital communities on the Internet with like-minded people. In a process of participatory sense-making, digital communities with their ideologies provide him with a shelter and a way to understand himself and the world. They also offer a remedy. Pre-existing personal grievance and decline are not only recognized but reframed into a new narrative of white supremacy. Affiliating with this narrative provides him with new ways of self-regulating feelings of insignificance and disconnectedness. The remedy it offers is to retaliate against the oppressor.

Roof is searching for meaning, for validation of his self-experience that is marked by grievance, and for possible solutions to his problems. He shapes the interaction with his environment by searching for stories and views similar to his. Human beings, like other biological species, generate meaning by interacting with their environment. The world ‘invites’ agents to use their creativity to design a world that is meaningful, valuable, and supportive. The bandwidth of this creative process is determined by the potential and capacities that are inherent to both subjects and the world. Roof’s autism spectrum disorder and the niche he co-constructed for himself both facilitate and limit this process of meaning creation.

Roof’s growing fascination and preoccupation with white supremacy ideologies help him to find and stabilize his own identity and to strengthen the existing identity of the group he is interacting with. This, possibly in combination with alcohol and drug use, leads to increasing agitation and the wish to do something about the threats to the white race. The fascination for the white supremacist view emerges after a prolonged period of almost complete isolation and of searching for avenues that could offer him a perspective. Influences that could have prevented him from action were weak or even absent. Roof’s condition was characterized by extreme social isolation and by a lack of opportunities to check his opinions with those of others, outside of his niche.^3^ As a result, alternative scenarios never could become real, ‘lived’ options. We are referring here to a combination of phenomena that is often seen in people with autism, i.e., mental rigidity, lack of imagination, and emptiness. Mental rigidity implies that there is only one interpretation and only one preferred course of action in a given situation. Lack of imagination implies that the person is unable to imagine alternative scenarios and/or that these scenarios never become real, ‘lived’ options. Emptiness refers both to the lack of inner resonance that autistic persons may experience in themselves and to what other people can experience in their interaction with autistic individuals. Resonance refers here to recognition of, and connection with, one’s affects and inclinations, background assumptions, and core values. People with autism are sometimes simply unable to allow alternative interpretations or plans to become ‘real’ enough to become something to consider. The alternatives remain empty options. All these factors—the agitation, the social isolation, and the possible disconnection with a deeper, resonating self—may have contributed to an unstable condition that made Roof even more sensitive to becoming enthralled by the relational dynamics developing between him and the extremist niche he co-constructed for himself. From an enactivist perspective, one could say that he enacted a world more and more separated from the common world around him, offering him new templates for meaning-making, new ways of making sense of and regulating himself that in turn afford more and more radical possibilities for action.

Consider the relational dynamics of Roof’s attempts to make sense of and regulate himself and his situation in interaction with his (online) environment as a trajectory (pathway) in a multi-dimensional state space of possible outcomes. Enactivism takes the possibility of his trajectory progressing in a non-linear way seriously: relatively small changes imposed on the system can lead to unexpected and disproportionately large behavioral changes, and vice versa, depending on the occurrent state of the system in relation to characteristics of its environment. Initially, several outcomes might be equally probable, but as time progresses the overall dynamics of the system might head toward a certain area in state space, making some outcomes more probable at the expense of others. Small perturbations might lead to a ‘tipping point’, after which even large changes applied to the system are not likely to change its course in state space, and only small nudges are needed to progress toward a particular outcome, such as violent extremism. This contributes to the unpredictability of lone actors’ behavior.

The criticized metaphor of radicalization as a conveyor belt (McCauley and Moskalenko, 2017) is indeed much too static to capture the dynamics of the path to (intended) violence. Even the analogy with a key/lock relationship, i.e., the relation between social exclusion and autistic rigidity, seems inadequate because, as the previous paragraphs suggest, in slightly different circumstances, a completely different combination of lock and key could have emerged. In typical cases, the emergence of a new order is nonlinear, in a way that cannot easily be predicted or derived from preceding causes. Non-linearity usually occurs in systems that are non-or minimally decomposable, i.e., in systems of which the parts cannot or can hardly be distinguished from one another, due to emergent phenomena shaping the conditions for their own existence, for example. The question whether in cases like Dylann Roofs the relationship between the interacting ‘parts’ and the new emerging order can be modeled as a nonlinear relationship, is ultimately an empirical matter and requires further scrutiny. We believe, however, that the sudden emergence of adherence to one ideology instead of one of the many others (and the characterization of his radicalization not being taken seriously by a friend as a tipping point) might indicate a form of non-linearity.

## Discussion

Enactivism is not the perfect solution to the question of how people progress toward attacks, rather, it emphasizes the unequivocal unpredictable nature of individual pathways as trajectories of sense-making in a multi-dimensional state space of possible outcomes. The merit of enactivism for the practice of managing radicalization and curbing extremist violence is that it allows us to see the ‘why’ and ‘how’ of these phenomena. Here, this involves understanding the nature and role of grievance from a first-person perspective, taking into account the specific details of the social niche a person has co-created for himself. This focus helps to discover where the opportunities lie for effective tailor-made interventions, which will hopefully be more effective than general repression or symptomatic (reactive) interventions.

To sum up, we believe enactivism can shed new light on the phenomenon of grievance-induced violence for a number of reasons.

First, enactivism invites us to look at a pathway toward violence as an ongoing process of active self-regulation and sense-making spiraling in certain directions.

The individuals at risk actively take part in co-creating niches which facilitate and constrain the range of possibilities for meaningful engagement with the world. The relative unpredictability of this nonlinear process, in which violent extremism is an emergent property, is a matter of principle, not a sign of empirical weakness. This does not mean that we should give up on assessing individuals at risk. It does mean, however, that such assessments will not be fruitful if pathways are described only from an external, third-person point of view and by solely focusing on the individual at risk. Rather, complementary to the traditional risk assessment methods, enactivism invites us to the very process of self-regulation and sense-making. By doing so, the clinician gets acquainted with the phenomenal life-world of the individual involved and will be better able to assess where this spiral of self-regulation and sense-making is headed. This can be done by paying attention to interaction patterns from an engaged perspective, making use of first-person and second-person narratives of the individuals involved.

Second, and in addition to the first, it is important to highlight violent behavior as the result of mutually constraining bottom-up and top-down forces, which reach beyond the individual agent. The process of radicalization is embodied, embedded, and extended in the wider socio-political environment. In other words, enactivism shows how national antiterrorism policy, or educational opportunities, contribute to individual sense-making, as do personal experiences and grievance. In the case description, the enactive account puts Roof’s fixation on his perceived bodily state into a framework of embodiment and embeddedness, for which he sought a niche that could serve as an affective scaffolding for feelings of inadequacy and inferiority. When the pathway, as described by Allely and Faccini (2019), is seen from an enactive angle, it also becomes apparent how (and not just ‘that’) his initial feelings of unease developed into his sense of self in dynamic interaction with his environment. Thus, we should take the agent-in-relation-to environment (niche) as our central unit of analysis, not the individual *per se*.

Third, enactivism suggests that grievance should not be understood simply as a particular interpretation or experience of one’s reality, indicated by certain psychological and social determinants. Enactivism puts forward a view in which grievance is the result of *a transformation in one’s relation towards the world,* which is fundamentally different from forming a (cognitivist) opinion *about* the world. Transformation involves an affectively charged change in self-organization that is related to changing interaction patterns (scaffolds) and ultimately may result in bringing radicalized ideas into practice. The literature on moral injury illustrates how pervasive and existential this transformation can be.

Fourth, the enactive approach is broadly applicable in a wide array of other areas such as biology, phenomenology, artificial life, social science, robotics, psychology, and neuroscience (Froese and Di Paolo, 2011). The essence is that the focus shifts from the individual to that what happens between the individual and his environment. Professionals assessing individuals at risk can also themselves become constitutive elements of that environment. Their sense-making activities can become an active element in a looping process that may lead to radicalization and to deradicalization.

Finally, in qualitative research on violent extremism, enactivism can help to avoid reinforcing stigmas and re-establishing a confirmation bias, by integrating the enactivist approach into cognition in established research methods, such as thematic analysis, focus group discussions, (non-) participatory observation, or interviews (Stilwell and Harman, 2021). By focusing more on the first-person perspective, we can build a picture of how people adapt to challenges in their lives. We claim that by exploring the dynamics of someone’s mindset in relation to his surroundings, the enactivist approach enables us to identify additional, more personal, and more effective ways to divert an individual pathway to violence, rather than having to use uniform policy strategies based on pejorative classifications like ‘terrorist’ or ‘lone actor’ that lack the broader explanatory context.

In practical terms, the enactive approach illustrates how pathways to extreme violence can become more meaningful when understood in terms of the five concepts of embodiment, experience, autonomy, sense-making, and emergence. The approach can be applied to a face-to-face encounter, but also to multi-disciplinary collaboration about an individual. There are some reports illustrating how enactivism can enhance the value of a psychiatric interview (Klin et al., 2003; De Jaegher, 2013; Glas, 2020), but more research is needed. One may think of recent computational approaches to psychopathology that combine computational modeling based on the principle of active inferencing (or: predictive coding) with evolutionary principles and socio-cultural analysis of looping effects (Adams et al., 2016; Friston et al., 2017; Wichers et al., 2019; Constant et al., 2020, 2022). This promising line of research is still in its infancy with respect to the clinical context. At the clinical level, there is also room for enactive reinterpretation of, for instance, emotions and moods (Lewis, 2005; Colombetti, 2014; Glas, 2020), developmental psychopathology (Klin et al., 2003; De Jaegher, 2013), and network approaches to clinical diagnosis and treatment (for instance Wigman et al., 2017).

As far as we know, there is no body of research on the use of the enactive approach in collaboration. We suggest, however, that this could be worthwhile to explore, especially in the context of structured professional judgment (SPJ). The aim would then be to investigate how enactivism can support a SPJ framework, in which risk factors are considered to assist clinical judgment. Again, this does not mean that the current procedures should be replaced by an enactive approach, but that the material available, when viewed from an enactive angle, can shed more light on the dynamics of the pathways to violence, like what Bouhana and Wikström (2010) refer to as the causes of causes. In the absence of the person who is the subject of the multi-disciplinary discussion, special attention is needed for maintaining a reflexive stance of all participants. In assessing to what extent, why, and how someone has radicalized, participants need to critically question the origin and development of their own ideas about the person being discussed in particular, and radicalization in general. Source material should be critically assessed, to avoid labeling interpretations as facts about the individual. Tunnel vision should be avoided by encouraging the exploration of alternative explanatory models.

Finally, the enactive approach can help in following up concerns which are raised about someone by, for example, a teacher, a doctor, or family. This should then lead to talking with, and not just about the individual, to gain insight into the dynamics of the behavior, bearing in mind the five concepts discussed above. The advantage of using this approach for individuals concerned, is that they can become aware of how their sense-making is related to earlier experiences and external influences. Apart from identifying specific opportunities for the management of people with radical ideas, the real bonus of an enactive approach would be for the individual to gain insight in this very process and the underlying personal needs and grievances.

## Author contributions

All authors listed have made a substantial, direct, and intellectual contribution to the work and approved it for publication.

## Conflict of interest

The authors declare that the research was conducted in the absence of any commercial or financial relationships that could be construed as a potential conflict of interest.

## Publisher’s note

All claims expressed in this article are solely those of the authors and do not necessarily represent those of their affiliated organizations, or those of the publisher, the editors and the reviewers. Any product that may be evaluated in this article, or claim that may be made by its manufacturer, is not guaranteed or endorsed by the publisher.

## Footnotes

^1^The self-organization involved in hierarchies of autonomous systems implies that top-down and bottom-up processes go together and are complementary. ‘Top’ refers to the whole and ‘down’ to the constituent parts. The idea is that subsystems act as interrelated components (or parts) that in virtue of their interaction develop emergent properties that constrain and enable new forms interactions between the parts, thereby possibly giving rise to new emergent properties, etc. The emergent properties cannot be inferred from the intrinsic properties of the components. It is in fact the other way around: the constant re-arrangement of the components defines both the components and the system. Thompson (2007) coins the term relational holism for this approach. Wholes and parts are defined by relations rather than by intrinsic properties of the components (subsystems).

^2^De Jaegher and Di Paolo conceive of social interaction on a spectrum of participation, ranging from sense-making that remains largely an individual activity at most modulated by the coordination in interaction, to a fully joint process of sense-making where the whole sense-making activity becomes a shared one and where meaning is created and transformed through patters of coordination. Importantly, for interaction to remain an instance of *social* interaction (rather than, say, coercion or mere physical interaction), the emergent autonomous organization in the relational dynamics should not completely destroy the autonomy and sense-making of the individual agents involved, although the scope of the latter may be augmented or reduced (De Jaegher and Di Paolo, 2007, p. 493).

^3^Roof’s ASD may have contributed to him being less in contact with a ‘deeper’, moral self, that could have provided him the intentions, inclinations, and reasons to abstain from violence and to search for an alternative. The term ‘deeper self’ refers to the ensemble of feelings, inclinations, ideas, values, and principles that help people to navigate through life, also in ambivalent and conflicting situations. However, there is controversy in literature over differences in morality between people with ASD and neurotypicals (Dempsey et al., 2020).
